# Beyond the Bowel: A Narrative Review Examining Bidirectional Mental Health Pathways Across Disorders of Gut-Brain Interaction and Inflammatory Bowel Disease

**DOI:** 10.7759/cureus.116451

**Published:** 2026-09-17

**Authors:** Akshata Gailot, Naser Banihani, Nazar Muhammad

**Affiliations:** 1 Internal Medicine, American University of the Caribbean School of Medicine, Philipsburg, SXM; 2 Surgery, American University of the Caribbean School of Medicine, Philipsburg, SXM; 3 Psychiatry, Cornerstone Family Healthcare, New York, USA

**Keywords:** anxiety, depression, gut-brain axis, gut-brain interaction, inflammatory bowel disease, irritable bowel syndrome, mental health, psychogastroenterology

## Abstract

Irritable bowel syndrome (IBS), functional dyspepsia, and inflammatory bowel disease (IBD) frequently coexist with psychological distress, but their assessment and treatment require distinct clinical interpretations. This narrative review examines how gut-brain evidence can guide psychiatric differentiation, prevent diagnostic overshadowing, and support coordinated care in adults with these conditions. A targeted PubMed/MEDLINE search through August 27, 2026 prioritized consensus reports, systematic reviews, randomized trials, longitudinal studies, and mechanistic reviews. Established neural and neuroendocrine communication is distinguished from proposed symptom-generating mechanisms, which often remain supported predominantly by preclinical evidence. Longitudinal associations between gastrointestinal illness and psychiatric outcomes do not establish individual causation. Assessment distinguishes gastrointestinal-specific fear from psychiatric disorders, screening symptoms from diagnoses, and fear-driven food restriction from eating disorders involving weight or shape concerns. Behavioral therapies and central neuromodulators may improve selected IBS outcomes, although certainty, tolerability, and access vary. Psychological treatment in IBD primarily addresses distress and functioning; inflammatory activity requires separate evaluation. The review proposes a clinical framework linking positive gastrointestinal diagnosis, targeted psychiatric assessment, shared treatment goals, and outcome-specific follow-up. Its contribution is a clinically focused synthesis rather than a validated algorithm or comprehensive systematic review. Incomplete original search documentation and heterogeneous evidence limit reproducibility and generalizability.

## Introduction and background

Gastrointestinal symptoms may be overlooked when attributed prematurely to anxiety or depression, while psychiatric symptoms may remain unrecognized during repeated gastrointestinal investigation. Concurrent gastrointestinal and mental health assessment may reduce this form of diagnostic overshadowing [1,2]. This review is intended for gastroenterologists, psychiatrists, primary care clinicians, and trainees caring for adults with irritable bowel syndrome (IBS), functional dyspepsia, or inflammatory bowel disease (IBD).

The Rome Foundation’s Rome V diagnostic and clinical framework describes disorders of gut-brain interaction (DGBIs) as conditions involving combinations of altered gastrointestinal motility, visceral sensitivity, mucosal and immune function, the gastrointestinal microenvironment, and central nervous system processing [1]. IBS and functional dyspepsia are DGBIs, whereas Crohn disease and ulcerative colitis are immune-mediated inflammatory disorders. In IBD, biopsychosocial assessment complements rather than replaces evaluation of intestinal inflammation [2].

Rome V updates diagnostic concepts and treatment approaches, reduces imprecise or stigmatizing terminology, and gives greater consideration to illness experience and sociocultural context. These developments support positive gastrointestinal diagnosis and targeted assessment of psychiatric and behavioral needs [1,2]. Whether this approach prevents diagnostic overshadowing or improves outcomes through mechanism-based treatment selection remains a subject for prospective evaluation.

The Rome Foundation Global Epidemiology Study included 73,076 adults from 33 countries. At least one Rome IV-defined DGBI was identified in 40.3% of Internet respondents (95% confidence interval [CI], 39.9-40.7) and 20.7% of household respondents (95% CI, 20.2-21.3) [3]. Sampling, geography, and survey method limit direct comparison between these estimates. A separate meta-analysis reported IBS prevalence of 9.2% using Rome III criteria (53 studies; 395,385 participants; 95% CI, 7.6-10.8; I²=99.7%) and 3.8% using Rome IV criteria (six studies; 82,476 participants; 95% CI, 3.1-4.5; I²=96.6%) [4]. These estimates predate Rome V and should not be interpreted as Rome V prevalence estimates.

A systematic review of 73 publications reported anxiety symptoms in 39.1% of patients with IBS (43 articles; 16,572 patients; 95% CI, 32.4-45.8; I²=99.1%) and depressive symptoms in 28.8% (47 articles; 22,842 patients; 95% CI, 23.6-34.0; I²=99.1%) [5]. The review included participants aged 15 years or older and used heterogeneous instruments and clinical settings. These screening-based estimates are not equivalent to rates of formally diagnosed psychiatric disorders. A 2026 Taiwanese administrative cohort identified longitudinal associations in both directions between IBS and diagnosed anxiety or depression [6], consistent with earlier Australian prospective findings [7]. Temporal ordering supports bidirectional associations but does not establish individual causation because ascertainment differences and residual confounding remain possible.

This review examines how gut-brain evidence can guide psychiatric assessment and treatment alongside gastrointestinal care in adults with IBS, functional dyspepsia, and IBD. It connects Rome V principles with psychiatric diagnostic differentiation, prevention of diagnostic overshadowing, condition-specific treatment selection, and coordinated follow-up. This clinically focused synthesis complements existing Rome reports and systematic reviews; the proposed framework has not yet been validated as a treatment-selection algorithm.

## Review

Literature search strategy

This narrative review addresses adult clinical care in IBS, functional dyspepsia, and IBD. A targeted PubMed/MEDLINE search covered literature available through August 27, 2026. The database offers relevant biomedical and clinical coverage across gastroenterology and psychiatry; reliance on a single database may nevertheless omit behavioral and psychiatric literature indexed elsewhere. The synthesis is selective and does not establish comprehensive coverage.

Search concepts combined “gut-brain axis,” “brain-gut axis,” and “disorders of gut-brain interaction” with mental health, anxiety, depression, psychiatric symptoms, psychological distress, and stress. Disease-specific searches addressed IBS, functional dyspepsia, and IBD. Additional concepts covered visceral sensitivity, autonomic and vagal signaling, the hypothalamic-pituitary-adrenal (HPA) axis, permeability, the microbiome, trauma, behavioral therapies, hypnotherapy, psychotherapy, neuromodulators, antidepressants, and multidisciplinary care. Rome reports, professional guidelines, systematic reviews and meta-analyses, randomized trials, longitudinal population studies, and contemporary mechanistic reviews were prioritized. Earlier publications were retained for foundational findings, and reference lists of key publications were examined. Human clinical evidence informed clinical interpretation; preclinical findings supplied mechanistic context. These are narrative selection priorities, not a prospectively registered eligibility protocol. Exact original queries, execution dates, search limits, screening counts, and reviewer-selection procedures are not established by the available search documentation. Supplementary Material 1 identifies these gaps and supplies a proposed Boolean strategy, explicitly distinguished from the original search.

Synthesis is organized by pathways, disorder-specific evidence, assessment, treatment, and integrated care. Appraisal considers design, measurement, bias, confounding, heterogeneity, precision, and applicability. Major numerical claims and recent references were checked during revision against available publisher records, indexed abstracts, and full texts; this was a targeted verification exercise, not an independent duplicate extraction. Published risk-of-bias judgments and certainty ratings are attributed to their source reviews. No new pooling, formal selection-flow diagram, independent study-level risk-of-bias assessment, or formal certainty grading was undertaken.

The review was revised with attention to the Scale for the Assessment of Narrative Review Articles (SANRA) [8]. A completed domain appraisal is provided in Supplementary Material 1. SANRA concerns narrative-review quality and does not establish search completeness or replace study-level bias assessment.

The gut-brain axis: an integrated bidirectional system

The gut-brain axis links the central nervous system and gastrointestinal tract through autonomic, vagal, spinal, enteric, neuroendocrine, immune, and sensory pathways. These interact with cognition, behavior, and the intestinal microenvironment [1,2,9]. Neural regulation of motility and secretion, visceral sensory transmission, and neuroendocrine stress responses are established physiology; their contribution to symptoms varies between patients.

Stress can influence gastrointestinal function and perception, while distension, nutrients, pain, and inflammation generate signals reaching central homeostatic and emotional networks. Specific barrier, immune, and microbial mechanisms remain supported substantially by preclinical and translational research, with variable human confirmation [9]. Associations with psychiatric symptoms may reflect diet, medications, disease severity, or other confounders. Established communication therefore does not identify the pathway maintaining an individual's symptoms. The major pathways involved in bidirectional gut-brain communication and their potential clinical relevance are summarized in Table 1.

**Table 1:** Major Components of Bidirectional Gut-Brain Communication DGBI: disorder of gut-brain interaction; IBD: inflammatory bowel disease. Evidence-status labels describe evidence type and maturity rather than formal certainty grades. Established physiology does not establish individual symptom causation or the efficacy of treatment targeting that pathway.

Pathway	Brain-to-gut influence	Gut-to-brain influence	Potential clinical relevance	Evidence status and limitations
Hypothalamic-pituitary-adrenal axis [9]	Neuroendocrine effects on gastrointestinal function.	Visceral and inflammatory effects on stress responses.	Stress-associated symptom exacerbation.	Established stress physiology; specific symptom-maintenance mechanisms remain substantially preclinical.
Autonomic nervous system [1,9]	Regulation of motility and secretion.	Afferent input to central autonomic networks.	Altered bowel function and nausea.	Established physiology; symptoms do not identify a specific autonomic abnormality.
Vagal pathways [1,9]	Gastrointestinal regulation and immune signaling.	Afferent visceral information.	Satiety, interoception, autonomic regulation.	Established neural communication; proposed immune and treatment applications require separate validation.
Enteric nervous system [1,9]	Extrinsic modulation of intrinsic circuits.	Enteric interaction with extrinsic sensory pathways.	Motility and sensory disturbances.	Established physiology; individual symptom attribution remains uncertain.
Visceral sensory pathways [2,10]	Cognitive, emotional, and descending modulation.	Distension and nociceptive input.	Hypersensitivity and persistent pain.	Human experimental and clinical evidence; group findings do not establish individual mechanisms.
Immune signaling [9,11]	Possible stress-related immune modification.	Inflammatory effects on central function.	Inflammatory disease and proposed DGBI mechanisms.	Established inflammation in IBD; stress-immune mechanisms in DGBIs have mixed preclinical and human support.
Intestinal barrier [9,11]	Possible stress-related permeability changes.	Luminal activation of immune pathways.	Proposed contribution in selected settings.	Mainly preclinical and translational causal evidence; human relevance varies.
Gut microbiome [9,11]	Stress and behavior may influence microbial ecology.	Microbial metabolites may act through neural, immune, endocrine, and metabolic pathways.	Research target requiring condition-specific clinical evidence.	Preclinical mechanisms and human associations; confounding and inconsistency limit clinical interpretation.
Cognition and behavior [2,10]	Fear, hypervigilance, and avoidance may influence symptom burden and disability.	Recurrent gastrointestinal symptoms may reinforce threat anticipation and avoidance.	Behavioral treatment targets.	Clinical, experimental, and observational support; a formulation, not a universal causal sequence.

Psychological stress and the hypothalamic-pituitary-adrenal axis

Psychological and physical stressors activate corticotropin-releasing hormone pathways and the hypothalamic-pituitary-adrenal (HPA) axis, which interact with autonomic and enteric signaling. Experimental studies link repeated stress to altered sensitivity, permeability, and immune activity, but many specific mechanisms derive from animal models and vary by stressor and duration [9]. Gastrointestinal symptoms and their anticipated consequences may themselves provoke anxiety and avoidance [2,10]. These observations do not establish psychogenic causation or demonstrate that HPA signaling maintains an individual’s symptoms [9,10].

Autonomic, vagal, and enteric nervous system signaling

Enteric circuits coordinate motility and secretion while interacting with sympathetic, parasympathetic, vagal, and spinal pathways. Vagal and spinal afferents convey visceral information to central homeostatic and sensory networks [1,9]. Nausea, urgency, and bowel changes may accompany autonomic responses, but these symptoms do not identify a specific autonomic abnormality in an individual patient [1,9]. Accordingly, this review distinguishes established physiological communication from the unproven attribution of an individual’s symptoms to a particular pathway; treatment efficacy requires separate clinical evidence.

Visceral hypersensitivity, interoception, and central sensory processing

Visceral hypersensitivity occurs in some patients with DGBIs and may reflect peripheral sensitization, spinal processing, or descending modulation [1,2]. Interoception, the perception and interpretation of internal signals, is influenced by attention, expectations, learning, and emotion. The psychobiological model connects these processes with gastrointestinal-specific fear and avoidance and proposes mechanisms that require further testing [10]. We therefore use the model as a clinical formulation rather than a proven causal sequence in every patient [10]. Group-level sensory or brain findings should not be assumed to identify an individual’s predominant mechanism; longitudinal and intervention studies are needed to establish mediators of improvement [2,10]. 

Intestinal barrier function, immune signaling, and the microbiome

Stress-related changes in permeability, immune activity, and microbial metabolism may interact with neural and endocrine pathways [9,11]. Specific causal mechanisms are better supported in animals than in humans. Human findings are affected by diet, transit, medications, geography, disease activity, and sampling and analytical methods. An associated microbial pattern therefore does not establish causation or provide an individual diagnosis. Microbiome-directed treatment requires condition-specific clinical evidence [11].

Sex, hormonal factors, and visceral pain

Several DGBIs are more prevalent among women, and experimental findings suggest interactions among reproductive hormones, stress, and visceral pain. Biological and psychosocial influences are interrelated, and experimental findings vary substantially between individuals; epidemiological differences therefore do not establish hormonal causation [12]. Clinical generalizability requires better characterization of menstrual phase, hormonal medications, age, and menopausal status, with representation across sexes and life stages [12].

Anxiety and gastrointestinal symptoms

Gastrointestinal-specific anxiety involves anticipated symptoms or consequences, such as urgency, vomiting, pain, or limited restroom access. It may occur with or without a formal anxiety disorder [2,10]. Assessment should distinguish this pattern from generalized worry across multiple life domains, recurrent unexpected panic attacks, and trauma-related symptoms. Screening scores identify symptom burden and require diagnostic follow-up; they do not independently establish generalized anxiety disorder, panic disorder, or post-traumatic stress disorder. The IBS prevalence estimates described in the Introduction are therefore not interchangeable with rates of diagnosed anxiety disorders [5]. Treatment targets depend on the formulation: gastrointestinal-focused behavioral treatment may address fear and avoidance, while a coexisting psychiatric disorder requires assessment and treatment of its broader symptoms [2].

Depression and gastrointestinal symptoms

Gastrointestinal and depressive symptoms may coexist and require assessment within a biopsychosocial framework [2]. The Taiwanese longitudinal study supports associations in both directions between IBS and diagnosed depression or anxiety, but administrative diagnoses, healthcare-contact patterns, and unmeasured confounding limit causal interpretation [6]. The publication reports different denominators and outcome presentations across analyses; its findings are therefore retained qualitatively here, without treating abstract-level hazard ratios as interchangeable with the combined outcomes in the main results [6]. Clinically, depressive symptoms require assessment of duration, pervasiveness, depressed mood, anhedonia, functional impairment, and suicidal thoughts. Fatigue, appetite change, and poor concentration should be interpreted within a complete assessment that also considers medical conditions, nutritional deficiencies, and substance-related explanations [13]. This approach supports differentiation of a depressive syndrome from overlapping physical symptoms while identifying safety concerns that require prompt attention [13].

Trauma-related symptoms and early adversity

A meta-analysis of eight observational studies, including 648,375 participants, associated post-traumatic stress disorder with higher odds of IBS (pooled odds ratio, 2.80; 95% CI, 2.06-3.54; I²=91.59%) [14]. Four studies were cross-sectional, and four were cohort studies; most involved US military veterans. High heterogeneity, differing ascertainment methods, and population selection limit generalizability and causal inference. Hyperarousal, sleep disturbance, and altered threat processing are possible explanatory pathways, rather than demonstrated mediators of the pooled association [14].

Separately, a meta-analysis of 15 studies involving 272,686 participants characterized the association between adverse childhood experiences and IBS as uncertain because of substantial heterogeneity (I²=93.58%) and evidence of publication bias. Differences in exposure and outcome ascertainment, study size, and study quality further complicate interpretation [15]. Childhood adversity and a current trauma-related disorder are distinct constructs. Assessment for post-traumatic stress disorder considers a qualifying traumatic exposure, intrusion, avoidance, negative changes in cognition or mood, arousal and reactivity symptoms, duration, clinically significant distress or impairment, and exclusion of substance-related or medical explanations; trauma exposure alone is insufficient [16]. These distinctions should guide the clinical interpretation of trauma histories in patients with IBS.

Irritable bowel syndrome as a model disorder of gut-brain interaction

IBS encompasses heterogeneous contributions from motility, visceral sensitivity, diet, postinfectious changes, mucosal signaling, and central processing [1,17]. A positive diagnosis rests on characteristic symptoms and appropriate assessment of clinically important alternatives [17]. Psychiatric or behavioral assessment can proceed concurrently when fear, avoidance, mood symptoms, or functional impairment affect care [2]. New alarm features require medical reassessment regardless of psychiatric history [17].

IBS does not itself establish somatic symptom disorder. That diagnosis requires distressing or disruptive somatic symptoms together with a persistent pattern of excessive symptom-related thoughts, feelings, or behaviors evaluated in clinical context. Apparently disproportionate concern, repeated consultations, or unrevealing investigations alone are insufficient to establish the full diagnosis [18]. Illness anxiety disorder instead centers on preoccupation with having or developing a serious illness, generally in the absence of substantial physical symptoms [18]. These distinctions help prevent psychiatric labeling based solely on symptom persistence or healthcare use.

Functional dyspepsia and mental health

Functional dyspepsia includes postprandial fullness, early satiation, epigastric pain, and epigastric burning, with possible contributions from altered accommodation, sensory processing, motility, and psychosocial factors. A systematic review of 13 observational studies involving 14,076 participants generally found greater anxiety and depressive symptom burden in functional dyspepsia, including refractory presentations [19]. Studies used different instruments, including the Hospital Anxiety and Depression Scale and Beck and Hamilton measures. Differences in diagnostic criteria, recruitment, and measurement limit comparability; the review did not provide a single pooled effect estimate [19]. These findings support targeted assessment but do not establish that anxiety or depression causes dyspepsia.

Food restriction requires assessment of its motivation and consequences. Restriction driven by anticipated pain, nausea, or vomiting differs from restriction driven by weight or shape concerns. Avoidant/restrictive food intake disorder (ARFID) warrants consideration when restricted intake causes significant weight loss, nutritional deficiency, dependence on oral nutritional supplements or enteral feeding, or marked psychosocial impairment [20]. When gastrointestinal disease coexists, the eating disturbance must exceed what is routinely associated with that condition and warrant additional clinical attention. Assessment should distinguish ARFID from medically appropriate dietary adjustment and from eating disorders involving weight or shape concerns [20].

Inflammatory bowel disease: a critical clinical contrast

Crohn disease and ulcerative colitis are immune-mediated inflammatory disorders. Evaluation of persistent symptoms includes assessment of inflammatory activity and, when indicated, infection, complications, medication effects, and nutritional deficiency. Mental health assessment complements this evaluation; psychological distress cannot determine whether inflammation is active.

A meta-analysis of 77 studies involving 30,118 patients reported anxiety symptoms in 32.1% (95% CI, 28.3-36.0; 58 studies; I²=96.9%) and depressive symptoms in 25.2% (95% CI, 22.0-28.5; 75 studies; I²=97.6%) [21]. These were questionnaire-defined symptoms, commonly measured using the Hospital Anxiety and Depression Scale. Active-disease groups had greater symptom burden, but disease activity was often classified using clinical indices rather than objective inflammatory measures. Differences in instruments, thresholds, geography, and disease activity limit application of these pooled estimates to individual settings. These symptom-based estimates cannot directly establish differences in rates of formally diagnosed psychiatric disorders between IBS and IBD [21].

Longitudinal findings also require qualification. A review of 12 studies involving 9,192 patients associated baseline anxiety or depression with several subsequent adverse IBD outcomes [22]. Evidence in the reverse direction was smaller: active IBD was associated with later anxiety symptoms in two studies involving 249 patients (relative risk (RR), 2.24; 95% CI, 1.25-4.01) and depression in three studies involving 642 patients (RR, 1.49; 95% CI, 1.11-1.98), with I²=0% for both analyses. The small evidence base limits reassurance from low statistical heterogeneity [22].

A separate review of 24 observational studies involving 630,408 patients associated depression with IBD flare (RR, 1.37; 95% CI, 1.16-1.63; I²=87.3%) [23]. Associations varied by disease subtype and baseline activity, and the total sample did not contribute to every outcome analysis. The flare association was not statistically significant in the subgroup of patients in remission at baseline. Disease severity, treatment exposure, adherence, and healthcare access may confound these relationships. The findings do not establish that treating depression prevents inflammatory relapse [23]. Gastrointestinal symptoms, objective inflammation, psychiatric symptoms, functioning, and quality of life therefore remain distinct assessment and treatment outcomes. The major mental health relationships and clinical implications across the gastrointestinal disorders discussed in this review are summarized in Table 2.

**Table 2:** Mental Health Considerations in Irritable Bowel Syndrome, Functional Dyspepsia, and Inflammatory Bowel Disease ARFID: avoidant/restrictive food intake disorder. Evidence-status labels are descriptive rather than formal certainty grades. Persistent pain, trauma-related symptoms, and avoidance are assessment considerations across these conditions rather than additional gastrointestinal diagnoses.

Gastrointestinal condition	Mental health relationship	Clinical implication	Evidence status and limitations
Irritable bowel syndrome [5,6,7,17]	Anxiety and depressive symptoms are common; longitudinal associations occur in both directions.	Assess gastrointestinal-specific fear and psychiatric disorders alongside the gastrointestinal diagnosis. Persistence or healthcare use alone does not establish somatic symptom disorder [16].	Observational evidence; heterogeneous measures and confounding limit causal inference. Screening symptoms differ from diagnosed disorders.
Functional dyspepsia [19,20]	Anxiety and depressive symptoms are frequent, including in refractory presentations.	Assess the motivation and nutritional consequences of food restriction; distinguish possible ARFID from weight- or shape-related restriction.	Observational evidence; diagnostic, recruitment, and measurement differences limit comparability and causal interpretation.
Inflammatory bowel disease [21-23]	Psychiatric symptom burden varies with activity and is associated with some adverse outcomes.	Assess inflammation and mental health separately while evaluating persistent symptoms.	Observational evidence with substantial heterogeneity and confounding. Associations vary by baseline activity and do not show that treating distress prevents relapse.

The gastrointestinal symptom-anxiety-avoidance cycle

Symptoms, threat anticipation, and avoidance may reinforce one another. For instance, after urgency during travel, a patient may monitor sensations and avoid journeys or meals. Immediate anxiety relief can limit opportunities to learn that symptoms are manageable. Cognitive behavioral therapy (CBT) may address avoidance and support restoration of activities [10,22]. This formulation should be assessed collaboratively, distinguishing practical precautions from disabling avoidance; it is not a universal causal sequence.

Clinical assessment at the gastroenterology-psychiatry interface

Consistent with the Rome V biopsychosocial framework, assessment should identify gastrointestinal-specific distress, psychiatric comorbidity, and behavioral barriers to recovery [2]. The Patient Health Questionnaire-9 and Generalized Anxiety Disorder-7 assess symptom burden and require diagnostic follow-up. Assessment should distinguish symptom-related fear from generalized anxiety or panic disorder, persistent depressive syndromes from overlapping fatigue or appetite changes, and trauma exposure from a current trauma-related disorder. Somatic symptom disorder requires its full clinical criteria; repeated consultations or apparently disproportionate concern alone are insufficient [18]. Food restriction warrants assessment of nutritional and psychosocial consequences, distinguishing fear of gastrointestinal symptoms and possible avoidant/restrictive food intake disorder (ARFID) from weight- or shape-motivated restriction [20].

Referral should follow identified needs, including disabling avoidance, psychiatric symptoms, nutritional compromise, or difficulty engaging with treatment. Suicidal ideation requires prompt risk assessment. New alarm features warrant medical reassessment regardless of psychiatric history. Gastrointestinal symptoms, psychiatric symptoms, and functional goals should be monitored separately.

A positive screening result should lead to assessment of symptom duration, impairment, psychiatric history, and alternative explanations. Gastrointestinal-specific distress can merit treatment even when criteria for a psychiatric disorder are not met. Conversely, diagnosing a psychiatric disorder requires assessment beyond the gastrointestinal complaint. Patient preference, cultural explanations of illness, and previous experiences of care should inform the shared formulation [2].

Communicating the gut-brain model without stigma

The American Gastroenterological Association recommends an empathic relationship, accessible explanations of pain mechanisms, and discussion of behavioral therapies and neuromodulators [25]. Clinicians should explain the positive gastrointestinal diagnosis, acknowledge uncertainty, and connect each intervention to an agreed target, such as pain, food-related fear, or disrupted sleep. An explanation such as “treatment addresses bowel function and the processing of bowel signals” can link the formulation to care without implying that symptoms are imagined.

Brain-gut behavioral therapies

Brain-gut behavioral therapies use structured techniques to address symptom-related cognitions, fear, attention, and avoidance [24]. A 2024 network meta-analysis included 42 randomized controlled trials (RCTs) and 5,220 adults with IBS. Compared with waiting-list control, self-guided or minimal-contact CBT reduced the risk of abdominal pain remaining unimproved (RR, 0.71; 95% CI, 0.54-0.95; network heterogeneity, τ²=0.0332). However, pain-response data were imputed from continuous outcomes in 29 trials, and no trial had low risk of bias across all domains [26].

A 2025 network meta-analysis of 67 RCTs involving 7,441 adults assessed global IBS symptoms. Compared with waiting-list control, the RR of non-improvement was 0.65 (95% CI, 0.53-0.80; nine trials, 1,150 participants) for CBT and 0.77 (95% CI, 0.61-0.96; five trials, 705 participants) for internet-based minimal-contact CBT; network heterogeneity was τ²=0.026. All comparisons had low or very low confidence. Differences in controls, treatment protocols, outcome definitions, and predominantly North American or European populations limit generalizability [27]. These overlapping reviews should not be treated as independent replications. Digital delivery may extend access, but efficacy is program-specific. Treatment selection should consider patient goals, accessibility, and preference; rankings do not establish clinical superiority.

Gut-directed hypnotherapy

Gut-directed hypnotherapy combines hypnotic induction with gastrointestinal-focused therapeutic suggestions. A 2025 review included 12 studies involving 1,158 patients; eight studies supplied continuous outcomes suitable for meta-analysis [28]. The pooled pain effect was small (standardized mean difference (SMD), 0.25; 95% CI, 0.01-0.49; I²=17%). The global-symptom estimate was imprecise and not statistically significant (SMD, 0.73; 95% CI, −0.09 to 1.55; I²=93%), with positive values favoring hypnotherapy [28]. Differences from other syntheses reflect varying outcomes, comparators, and inclusion criteria. Group or remote delivery may improve access, but these formats require evaluation within their specific protocols.

Central neuromodulators and psychopharmacology

Central neuromodulators are medications used to influence pain processing and other gut-brain functions; antidepressants constitute one group within this broader category [29]. The indication should specify gastrointestinal symptom modulation, treatment of a psychiatric disorder, or both. A 2025 meta-analysis included 28 placebo-controlled RCTs involving 2,475 adults with IBS. Tricyclic antidepressants (TCAs) had moderate-certainty evidence for global symptoms: the RR of non-improvement was 0.70 (95% CI, 0.62-0.80; 11 trials, 1,144 participants; I²=20%). Evidence for other drug classes and endpoints was less certain, and withdrawals because of adverse effects were more frequent with neuromodulators than placebo [29].

A 2026 network meta-analysis included 68 RCTs involving 6,694 participants [30]. Its main network contained 66 trials and 6,542 participants, combining trials reporting global symptoms or abdominal pain; it did not require improvement in both. Against waiting-list control, the RR of non-improvement was 0.49 (95% CI, 0.32-0.75; six trials, 387 participants) for serotonin-norepinephrine reuptake inhibitors (SNRIs) and 0.60 (95% CI, 0.43-0.85; 15 trials, 1,519 participants) for TCAs; network heterogeneity was τ²=0.044. These estimates incorporate indirect comparisons and do not demonstrate SNRI superiority over TCAs or behavioral therapies. No SNRI trial had low risk of bias, and most network comparisons had low or very low certainty [30].

For gastrointestinal neuromodulation, amitriptyline may be started at 10 mg nightly and titrated according to response and tolerability; doses may remain below those required for major depression [29]. TCAs can suit pain with diarrhea but may worsen constipation and cause dry mouth, sedation, orthostatic symptoms, or cardiac conduction effects. Selective serotonin reuptake inhibitors (SSRIs) and SNRIs have less consistent IBS-specific evidence and may be selected when a depressive or anxiety disorder independently warrants treatment. Their doses should follow the psychiatric indication and individual agent; an effective low-dose TCA regimen cannot be extrapolated to these classes. SSRIs can cause nausea, diarrhea, activation, and sexual dysfunction; SNRIs can cause nausea, sweating, sexual dysfunction, and blood-pressure elevation. SSRIs and SNRIs may also produce discontinuation symptoms. Medication review, monitoring, and gradual discontinuation should be individualized [29].

Psychological treatment in functional dyspepsia

A meta-analysis of nine RCTs involving 786 adults found possible benefits of psychotherapy for functional dyspepsia [32]. Post-treatment effects favored psychotherapy for symptom severity (Hedges’ g, 0.56; 95% CI, 0.09-1.03; I²=83%) and anxiety (g, 0.77; 95% CI, 0.18-1.36; I²=89%), each based on eight comparisons. Psychiatric outcomes reflected symptom scales rather than diagnostic remission. Small studies, diverse interventions, and substantial risk of bias limit certainty; the symptom-severity estimate crossed the null after adjustment for possible publication bias [32]. Treatment may be considered for persistent symptoms or disabling fear, but neither the optimal intervention nor the magnitude of clinically meaningful benefit is established.

Psychological treatment in inflammatory bowel disease

A 2023 meta-analysis included 25 RCTs, all at high risk of bias [33]. Among patients with predominantly quiescent IBD, psychological therapy produced small improvements at treatment completion in anxiety scores (13 trials, 1,088 participants; SMD, −0.23; 95% CI, −0.36 to −0.09; I²=18%) and depression scores (15 trials, 1,189 participants; SMD, −0.26; 95% CI, −0.38 to −0.15; I²=2%). These changes do not establish remission of psychiatric disorders. Relapse was not significantly reduced (10 trials, 861 participants; RR, 0.83; 95% CI, 0.62-1.12; I²=60%), and no significant improvement in disease-activity indices was demonstrated. Only four trials recruited active IBD populations, limiting conclusions in that setting [33]. Psychological treatment can address distress and functioning alongside anti-inflammatory treatment; disease-activity indices should not be equated automatically with objective biochemical or endoscopic inflammation.

Multidisciplinary integrated care

The Australian MANTRA trial randomized 188 adults with Rome IV-defined functional gastrointestinal disorders to gastroenterologist-only or multidisciplinary care [34]. Among 144 participants in the modified intention-to-treat analysis, global symptom improvement occurred in 84% with multidisciplinary care and 57% with gastroenterologist-only care (RR, 1.50; 95% CI, 1.13-1.93). The open-label, single-center design, post-randomization exclusions, and bundled intervention limit generalizability and attribution to individual disciplines. At 12 months after treatment, benefits varied by outcome; between-group differences in overall symptom improvement and combined anxiety/depression scores were not statistically significant [35].

Integrated services should match gastroenterology, psychological or psychiatric care, dietetics, and physiotherapy to identified needs. Implementation requires clear referral criteria, communication between clinicians, and attention to workforce, funding, and access [36]. The principal mental health and brain-gut interventions relevant to gastrointestinal care, together with their potential clinical roles and major limitations, are summarized in Table 3.

**Table 3:** Mental Health and Brain-Gut Interventions Relevant to Gastrointestinal Care CBT: cognitive behavioral therapy; DGBI: disorder of gut-brain interaction; IBD: inflammatory bowel disease; IBS: irritable bowel syndrome; RCT: randomized controlled trial; SNRI: serotonin-norepinephrine reuptake inhibitor; SSRI: selective serotonin reuptake inhibitor; TCA: tricyclic antidepressant. Formal certainty or confidence ratings reproduce those reported by the cited syntheses; other labels describe evidence type and limitations. No new formal grading was performed. Ratings concern the specified gastrointestinal populations and outcomes and do not assess efficacy across all indications, including established psychiatric uses.

Intervention	Principal target	Potential clinical role	Evidence status or reported certainty	Major limitations or harms
Psychoeducation [24,36,37]	Understanding and engagement.	Explain the diagnosis and agree on treatment goals.	Consensus-supported; independent efficacy difficult to isolate.	Often insufficient alone for substantial disability.
Gastrointestinal-focused CBT [24,26,27]	Fear, hypervigilance, avoidance, coping.	Persistent IBS symptoms and disability.	IBS RCT support; low or very low confidence in 2025 network comparisons.	Variable controls and protocols, limited blinding, and differences in access.
Exposure-based treatment [10,24]	Symptom-related fear and avoidance.	Graded exposure within individualized CBT.	Component of some CBT protocols; independent contribution less certain.	Requires appropriate medical and nutritional assessment.
Gut-directed hypnotherapy [26-28]	Symptoms and related distress.	Selected patients with IBS.	IBS trial evidence; small pooled pain benefit and an imprecise, nonsignificant global estimate in the 2025 review.	Heterogeneous protocols and outcomes; access varies.
TCAs [29,30]	Gastrointestinal pain modulation.	IBS neuromodulation: start low and titrate to response and tolerability.	Moderate certainty for global IBS symptoms in the 2025 placebo-controlled meta-analysis; certainty varies by outcome.	Constipation, dry mouth, sedation, orthostatic symptoms, and cardiac conduction effects; gastrointestinal doses may not treat depression.
SSRIs [29,30]	Coexisting anxiety or depression; possible neuromodulation.	Use and dose according to the psychiatric indication and individual clinical profile.	Less consistent IBS evidence; relevant estimates have low or very low certainty.	Nausea, diarrhea, activation, sexual dysfunction, and discontinuation symptoms.
SNRIs [29-31]	Coexisting anxiety or depression; possible pain modulation.	Use and dose according to the psychiatric or pain indication; monitor tolerability.	Limited IBS trials; low or very low certainty in most network comparisons and no SNRI trial at low risk of bias. Rankings do not establish superiority.	Nausea, sweating, sexual dysfunction, blood-pressure elevation, discontinuation symptoms.
Psychotherapy for functional dyspepsia [32]	Symptoms, distress, coping.	Persistent symptoms or disabling fear.	Limited RCT evidence; high heterogeneity and bias. Symptom benefit became nonsignificant after publication-bias adjustment.	Optimal intervention and clinical effect remain uncertain.
Psychological treatment in IBD [33]	Distress, adjustment, functioning.	Adjunct to indicated gastrointestinal treatment.	Small short-term psychological symptom-score benefits, mainly in quiescent IBD; all RCTs at high risk of bias.	Relapse reduction was not demonstrated; limited active-disease evidence. Symptom-score improvement does not establish control of inflammation.
Multidisciplinary care [34-36]	Overlapping gastrointestinal, behavioral, dietary, and psychiatric needs.	Complex or persistent DGBIs.	Single-center open-label RCT supports short-term benefit; longer-term differences vary by outcome.	Post-randomization exclusions, bundled treatment, resource requirements, and limited generalizability.

A practical integrated clinical framework

The proposed framework translates the Rome V biopsychosocial approach and the evidence reviewed here into practical assessment and coordinated care for IBS, functional dyspepsia, and IBD [1,2,37]. It represents a clinical synthesis rather than an independently validated treatment-selection algorithm.

Gastrointestinal and psychiatric assessment can proceed concurrently. For suspected IBS or functional dyspepsia, establish a positive diagnosis using appropriate clinical criteria and targeted evaluation of alarm features and relevant alternatives. In IBD, assess inflammatory activity, complications, medication effects, and nutritional status as indicated. Explain the diagnosis and connect each proposed intervention to an agreed symptom or functional goal. Assessment should identify gastrointestinal-specific fear, avoidance, mood symptoms, sleep disturbance, restrictive eating, and functional impairment. Screening results require diagnostic follow-up when clinically indicated. Symptom-related distress should be distinguished from a formal psychiatric disorder, and food restriction should be evaluated for nutritional consequences and possible ARFID [2,20]. New alarm features require clinically indicated medical evaluation regardless of psychiatric history.

Treatment selection should reflect the clinical formulation, condition-specific evidence, tolerability, availability, and patient preference. Disabling fear and avoidance may support gastrointestinal-focused CBT with graded exposure, while persistent IBS symptoms may support CBT or gut-directed hypnotherapy. A central neuromodulator may be considered for persistent pain, with its gastrointestinal or psychiatric indication explicitly defined. Psychological treatment for functional dyspepsia requires discussion of its less certain evidence base. Dietary intervention and specialist input should address identified needs, with coordinated care for complex presentations [24,25,29,32,36].

Follow-up should assess gastrointestinal symptoms, psychiatric symptoms, functioning, and treatment harms separately, together with objective inflammatory measures when indicated in IBD. Inadequate progress should prompt review of treatment exposure, tolerability, the clinical formulation, and access barriers. New alarm features or deterioration warrant medical reassessment. Selecting treatment around clinically identified targets does not establish the biological mechanism responsible for an individual’s symptoms.

Barriers to integrated gastroenterology and mental health care

Stigma, limited interdisciplinary training, shortages of clinicians skilled in brain-gut behavioral therapies, and fragmented funding and referral systems restrict access to integrated care [36]. Practical responses include clear referral criteria, a designated care coordinator, shared treatment goals, and explicit responsibilities for follow-up. Training should improve gastroenterologists’ recognition of psychiatric and behavioral needs and mental health clinicians’ understanding of gastrointestinal disorders.

Group treatment, telehealth, and remotely delivered behavioral interventions may broaden access, but their effectiveness depends on the specific program and population studied [24,27,28]. Implementation should address affordability, language needs, digital literacy, and access to technology, with pathways for specialist assessment when psychiatric or nutritional needs exceed the service’s capacity.

Limitations of the current evidence

This selective PubMed/MEDLINE-based narrative review may omit relevant literature. The original search cannot be reproduced from the reported concepts, and no independent risk-of-bias assessment or new quantitative synthesis was undertaken. Completeness cannot be assured.

Psychiatric ascertainment, gastrointestinal criteria, recruitment, and disease-activity definitions vary. Confounding limits observational causal inference; comparator choice, limited blinding, small studies, publication bias, and imprecision affect treatment estimates. Harms and durability also require consideration. Overlapping meta-analyses are not independent replications, and total samples may not contribute to every outcome. Statistical significance does not establish clinical importance. Table labels distinguish evidence type from published certainty ratings without implying a uniform grading system. Findings principally concern IBS, functional dyspepsia, and IBD in the studied populations and should not be extrapolated automatically to other conditions or age groups.

Future directions

Prospective studies should evaluate whether treatment selection based on gastrointestinal-specific fear, avoidance, psychiatric diagnosis, pain characteristics, or disease activity improves outcomes beyond usual care. Repeated measurements could clarify temporal relationships and candidate mediators of improvement. Prespecified analyses should examine sex, hormonal state, and relevant social factors [10,12]. Proposed physiological or microbial biomarkers require independent replication and evidence that they improve clinical prediction beyond standard assessment.

Comparative and combination-treatment trials should use appropriate active controls, clearly defined populations, patient-important outcomes, and systematic reporting of harms, adherence, and durability. Such studies are needed to determine whether combining pharmacological and behavioral treatments provides additional benefit; network rankings alone cannot resolve this question [27,31].

Pragmatic evaluations of integrated and remote care should assess engagement, workforce requirements, cost-effectiveness, and equitable access [36]. Gastrointestinal symptoms, psychiatric symptoms or diagnoses, functioning, quality of life, and healthcare utilization should be measured as distinct outcomes. In IBD, studies should additionally distinguish clinical symptom indices from biochemical or endoscopic inflammation [33]. This approach would clarify which interventions improve particular outcomes and which patients are most likely to benefit.

## Conclusions

In adults with IBS, functional dyspepsia, and IBD, gut-brain evidence supports coordinated gastrointestinal and mental health assessment. Clinical interpretation must distinguish established physiology from proposed causal mechanisms, psychiatric symptoms from diagnosed disorders, and gastrointestinal symptom relief from control of intestinal inflammation. Treatment can address gastrointestinal-specific fear, psychiatric comorbidity, pain, and functional impairment according to the condition-specific evidence and patient goals. Behavioral therapies and neuromodulators offer options for selected patients, with uncertainty concerning comparative efficacy, durability, and access. The proposed clinical framework provides an evidence-informed synthesis for practice; prospective validation and more transparent, reproducible evidence synthesis remain necessary.
